# Non-Engineered Nanoparticles of C_60_

**DOI:** 10.1038/srep02094

**Published:** 2013-06-28

**Authors:** Shigeru Deguchi, Sada-atsu Mukai, Hide Sakaguchi, Yoshimune Nonomura

**Affiliations:** 1 Institute of Biogeosciences, Japan Agency for Marine-Earth Science and Technology (JAMSTEC), 2-15 Natsushima-cho, Yokosuka 237-0061, Japan; 2 JST, ERATO, Akiyoshi Bio-Nanotransporter Project, Kyodai Katsura Venture Plaza, 1-36, Goryo Oohara, Nishikyou-ku, Kyoto 615-8245, Japan; 3 Department of Polymer Chemistry, Graduate School of Engineering, Kyoto University, Kyoto daigaku-Katsura, Nishikyo-ku, Kyoto 615-8510, Japan; 4 Deep Sea Engineering Science Program, Application Laboratory, JAMSTEC, 3173-25 Showa-machi, Yokohama 236-0001, Japan; 5 Department of Biochemical Engineering, Graduate School of Science and Engineering, Yamagata University, 4-3-16 Jonan, Yonezawa 992-8510, Japan

## Abstract

We discovered that rubbing bulk solids of C_60_ between fingertips generates nanoparticles including the ones smaller than 20 nm. Considering the difficulties usually associated with nanoparticle production by pulverisation, formation of nanoparticles by such a mundane method is unprecedented and noteworthy. We also found that nanoparticles of C_60_ could be generated from bulk solids incidentally without deliberate engineering of any sort. Our findings imply that there exist highly unusual human exposure routes to nanoparticles of C_60_, and elucidating formation mechanisms of nanoparticles is crucial in assessing their environmental impacts.

Nanoparticles, fine particles having length ranging from 1 to 100 nanometres in two or three dimensions^1^, exhibit properties that are not observed for molecules or bulk counterparts^2^. They are widely used as building blocks for nanotechnology-derived applications such as single-electron devices, ultra dense recording media, bioelectronic devices and sensors, bioimaging, optoelectronic devices, catalysis and chemical sensors, and energy conversion and storage^2^,^3^. Such ultrafine particles are usually prepared in a bottom-up manner by allowing molecules or atoms to assemble and build up into nanoparticles through chemical reactions in solutions or gas^2^,^3^.

Fine particles can also be obtained in a top-down manner by pulverising bulk solids^4^. When physical forces are applied to a solid, it undergoes plastic deformation to a breaking point, above which fracture results. The size of the solid is reduced as fracturing is repeated during pulverisation. However, as the size becomes smaller, the applied energy is rather dissipated as heat and the size reduction becomes increasingly difficult. Consequently, conventional milling devices typically produces particles with an average size no smaller than several ten micrometres^5^, and nanoparticles are not obtained unless very high energy is applied using a special device such as a high-energy ball mill^6^,^7^. We discovered entirely different size-reduction characteristics for bulk solids of fullerene C_60_.

## Results

**Figure 1:**
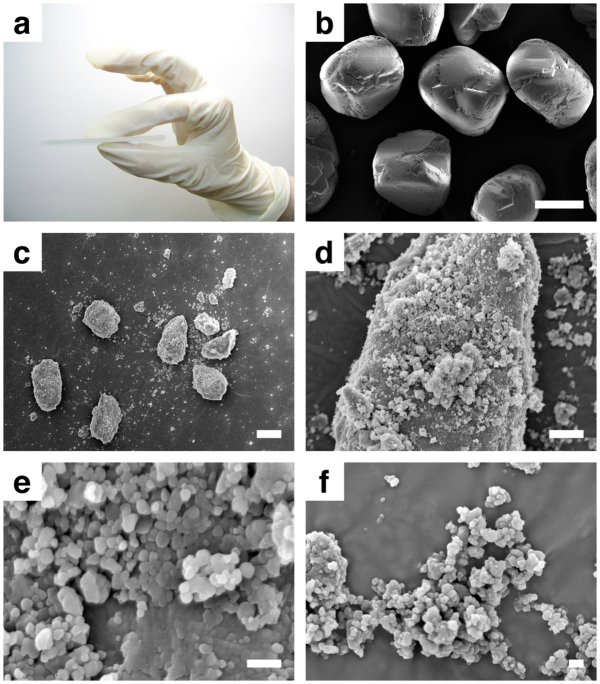
Formation of nanoparticles by rubbing solid C_60_ between glass slides. (a) Experimental procedure. (b) SEM image of particles of as-received solid C_60_. Scale bar represents 100 μm. (c, d, e and f) SEM images of nanoparticles of C_60_ formed by rubbing bulks solids between glass slides. Scale bars represent 10 μm (c), 2 μm (d), and 200 nm (e and f).

Solid C_60_ (1.5 mg) was placed between two glass microscope slides (76 mm × 26 mm, thickness 0.8 – 1.0 mm) and rubbed repeatedly between fingertips for a few minutes (Fig. 1a). Frictional resistance of C_60_ increased progressively as it was rubbed, and coarse black particles (Fig. 1b, median diameter 129 μm)^8^ eventually turned to fine brownish powder and adhered to the glass surface.

Examination by scanning electron microscopy (SEM) revealed that rubbed C_60_ had a bimodal size distribution, consisting of particles no larger than a few tens of micrometres and significantly smaller ones (Fig. 1c). Remarkably, detailed examination of the smaller particles showed that nanoparticles, some of which were even smaller than 100 nm, were generated by this mundane treatment. Such nanoparticles were mostly found associated on the surface of large particles (Figs. 1d and 1e), but agglomerates of nanoparticles were also observed around large particles (Fig. 1f).

**Figure 2:**
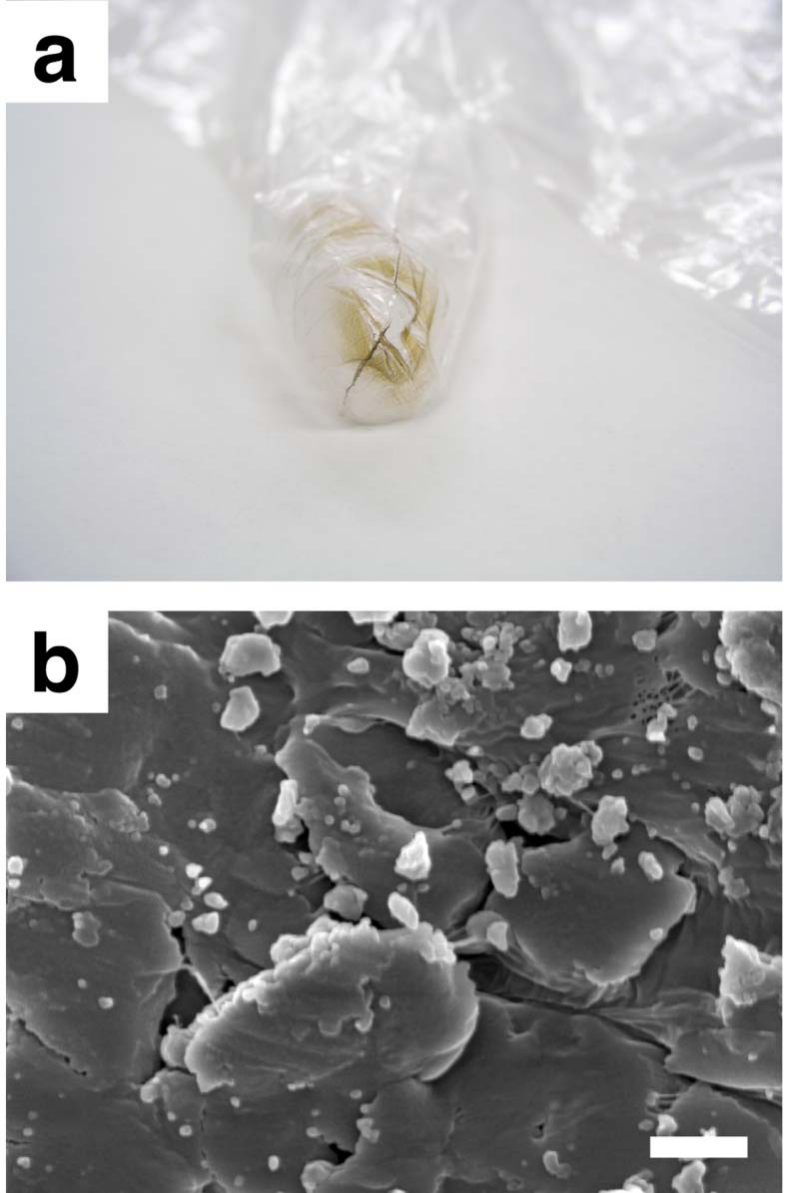
Formation of nanoparticles by rubbing solid C_60_ between fingertips. (a) A photograph showing C_60_ on the surface of the glove after rubbing. (b) SEM images of rubbed C_60_ on the glove surface. Particles that are smaller than 100 nm are seen. Scale bar represents 500 nm.

Use of the glass slides was not critical. When solid C_60_ (ca. 2 mg) was rubbed between fingertips without using the glass slides for 1 minute (a polyethylene glove was used to avoid direct contact of C_60_ with skin), nanoparticles smaller than 100 nm (Fig. 2b) were also observed in rubbed C_60_ on the surface of the glove (Fig. 2a), although chances of finding such nanoparticles was much less compared with C_60_ rubbed with the glass slides.

To quantify the size reduction by rubbing, C_60_ on the glass slides was dispersed in 2 mL of water containing 1 wt% of an anionic surfactant, sodium dodecyl sulphate (SDS). When the aqueous solution of SDS was poured onto rubbed C_60_ on the slides with a pipette, a brown and turbid dispersion was immediately formed. The dispersion was subjected to ultrasonic treatment for 5 min (Model 5510, 42 kHz output frequency, Branson Ultrasonic Corporation, Danbury, USA), and filtered with a membrane filter (nominal pore size, 5 μm). It should be noted here that ultrasonic treatment only helped disintegrating agglomerated nanoparticles and did not induce further size-reduction^9^.

**Figure 3:**
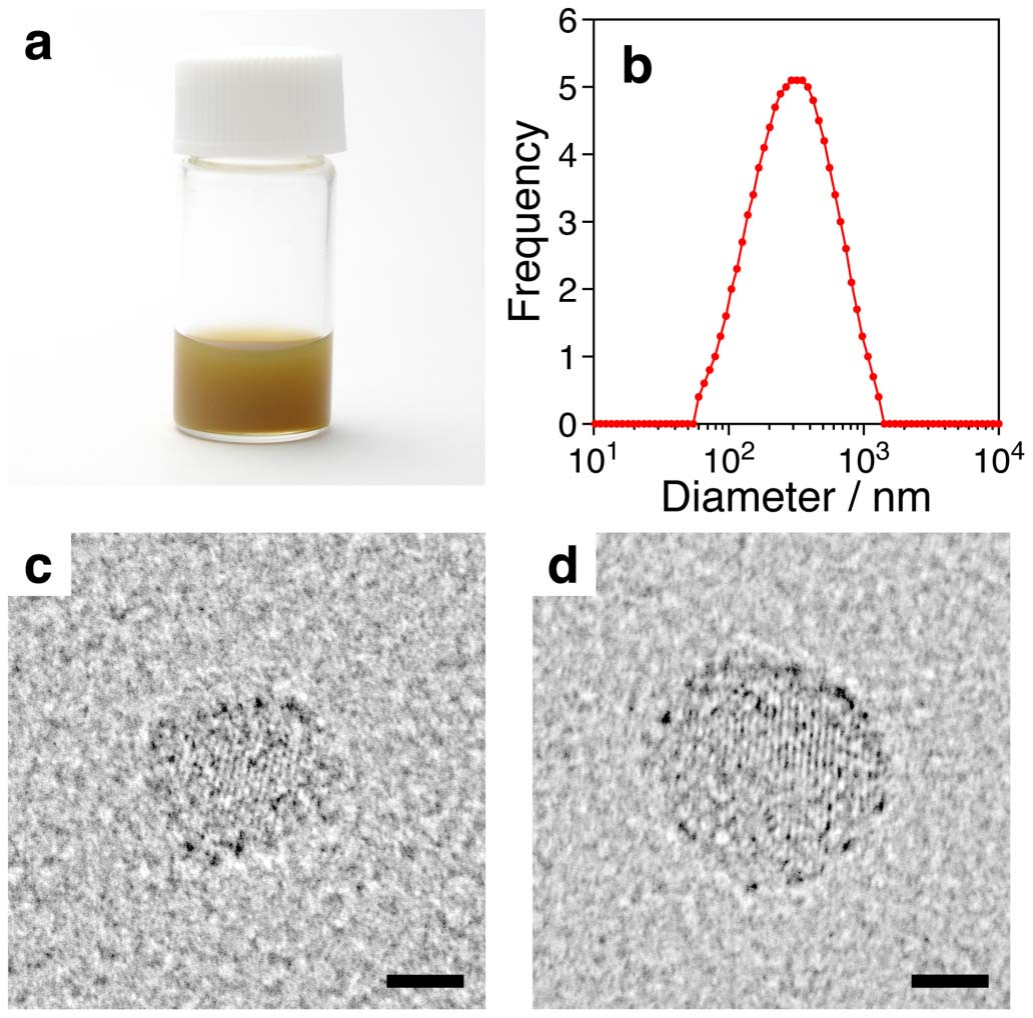
Size distribution of nanoparticles produced by rubbing solid C_60_ between glass slides. (a) An optical photograph of a dispersion of nanoparticles of C_60_ in water containing 1 wt% SDS. (b) Size distribution of the nanoparticles of C_60_ in the dispersion. (c and d) HRTEM images of nanoparticles of C_60_ found in the dispersion. Scale bars represent 5 nm.

The highly turbid and brown filtrate (Fig. 3a) was found to contain nanoparticles of C_60_, whose average diameter was 256.8 ± 1.1 nm by dynamic light scattering (Fig. 3b). The concentration of C_60_ in the dispersion (Fig. 3a), which was measured spectrophotometrically^10^, was 2.46 ± 0.17 × 10^−4^ M (177 ± 12 μg/mL), meaning that approximately 24 wt% of C_60_ solids was turned to particles smaller than 5 μm simply by rubbing them between fingertips. Examination by high-resolution transmission electron microscopy (HRTEM) revealed the presence of particles smaller than 20 nm in the dispersion (Figs. 3c and 3d). The particle in Fig. 3c had a dimension 9 nm × 12 nm, and the nearly spherical particle in Fig. 3d was 14 nm in diameter. Mean fringe spacing of both particles was 0.50 nm, and agreed well with the value for C_60_ crystal of (220) plane (0.50074 nm)^11^, showing that these particles are nano-sized crystals of C_60_ having an face-centred cubic (fcc) structure. Calculation using crystallographic data indicates that the spherical particle in Fig. 3d consists of approximately 2500 C_60_ molecules and 46% of them are exposed to the particle surface^8^. Rubbed C_60_ on the surface of the glove (Fig. 2a), on the other hand, could not be analysed in this way. C_60_ strongly adhered to the glove surface, and could not be removed and dispersed in water containing SDS.

Several unconventional engineering can produce nanoparticles of C_60_ from bulk solids. These include sonication of solid C_60_ in water^12^, prolonged stirring of solid C_60_ in water for several weeks^13^, or hand-grinding of solid C_60_ with an agate mortar and pestle^8^,^9^,^14^,^15^. Nevertheless, formation of nanoparticles by such a mundane treatment as rubbing between fingertips is unprecedented and noteworthy. Moreover, efficiency of size-reduction by rubbing between fingertips appears to be comparable to that of hand-grinding with an agate motor and pestle, by which approximately 34 wt% of C_60_ was turned to particles smaller than 5 μm^8^,^9^.

**Figure 4:**
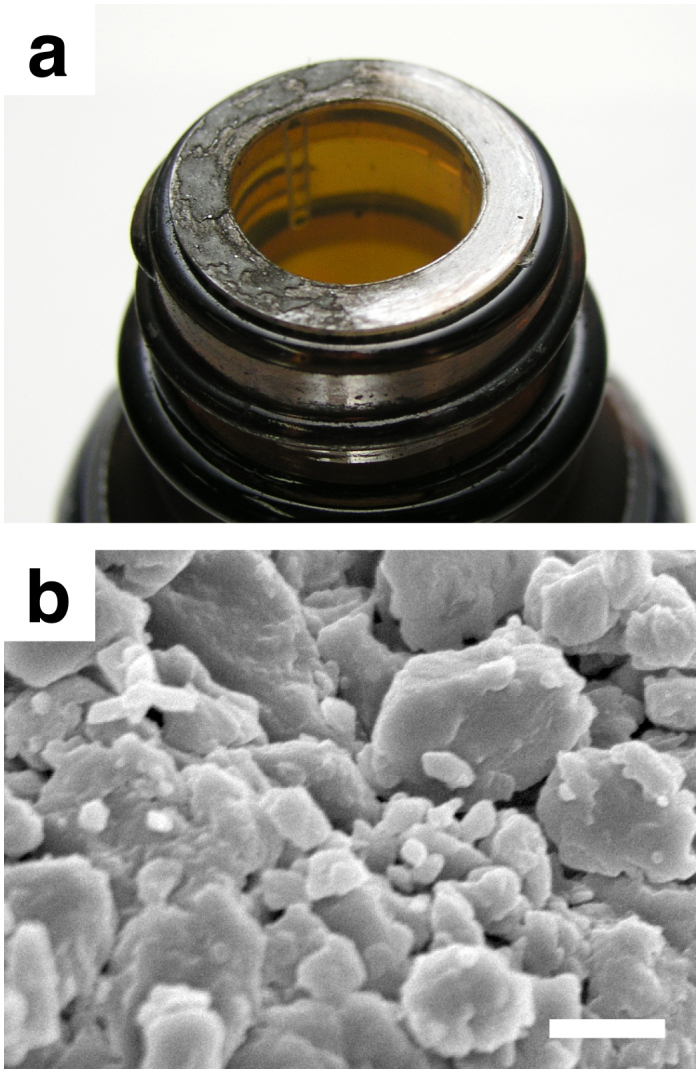
Non-engineered nanoparticles of C_60_. (a) A photograph showing a white bushing of the month of a reagent bottle of C_60_. Right-hand side of the bushing was cleaned before taking the picture to reveal the original white colour. (b) SEM image of C_60_ that was collected from the bushing. Nanoparticles that are smaller than 100 nm are clearly seen. Scale bar represents 500 nm.

Comparable size-reduction characteristics observed for both hand-ground and finger-rubbed C_60_ strongly suggests that top-down fabrication of nanoparticles of C_60_ by mechanical means require a surprisingly little effort. Far from it, we found that nanoparticles of C_60_ were generated even without deliberate engineering of any sort. Fig. 4a shows a mouth of a reagent bottle of C_60_. A white plastic bushing at the mouth was covered with brown powder of spilt C_60_, which had been compressed and sheared repeatedly by a screw cap whenever the bottle had been opened and closed. Examination by SEM revealed particles smaller than 100 nm in C_60_ that was collected from the bushing (Fig. 4b).

## Discussion

Considering the efforts usually required to prepare the nanoparticle by pulverisation^6^,^7^, the size-reduction characteristics of C_60_ are truely anomalous. The size-reduction process of solid C_60_ is purely physical, and is not associated with change in crystalline structure or chemical/mechanochemical reactions^8^,^9^. Formation of nanoparticles of C_70_ by pulverisation was also reported^16^, suggesting that anomalous size-reduction is a common characteristics among fullerenes. Detailed mechanisms behind the anomaly are not clear at present, but it could be ascribed to inherent properties of crystalline C_60_ such as fast isotropic rotation of the molecule^17^ or low cohesive energy (1.6 eV)^8^,^9^,^18^. Labille et al. used X-ray diffraction to study nanoparticles of C_60_ that were prepared by prolonged stirring of bulks solids in water, and proposed that nanoparticles of C_60_ are formed by an erosion of large C_60_ crystals, which occurs preferentially via (111) lattice plane exfoliation^19^. The adhesion energy between smooth C_60_ surfaces is an order of magnitude lower than that of typical van der Waals solids^20^. This is ascribed to structural features of the C_60_ molecule including a large size, rigidity and smooth surface, which makes the C_60_ molecule behave like a macroscopic spherical object rather than a conventional molecule^20^. Shear stress exerted by fingertips or a screw cap may be sufficient enough to overcome the low adhesion energy to induce exfoliation.

Concerns have emerged on possible adverse effects of engineered nanomaterials^21^, and nanoparticles of C_60_ have attracted considerable scientific attention in this regard^22^,^23^,^24^. Pulverization has widely been used to prepare C_60_ nanoparticles for evaluating their possible environmental and health impacts^25^. Compared with conventional methods for producing C_60_ nanoparticles such as recrystallisation from organic solvents (toluene^26^ or tetrahydrofuran^10^), pulverisation has distinct advantages that the product is free from residual solvents and the procedure is simple to perform. A major drawback of pulverisation is a broad size distribution of the nanoparticles produced, but this can be circumvented by size-fractionation using filtration or centrifugation^8^,^15^. The size of pulverised nanoparticles can also be controlled when an automated milling device (wet grinding using a bead mill) is employed^27^.

Present study shows that anomalous size-reduction characteristics of solid C_60_ have immediate implications for assessing health risks of C_60_ nanoparticles. Assessing possible human exposure routes to nanoparticles is an important consideration^21^,^28^, and is among five grand challenges necessary toward safe handling of nanotechnology^29^. As nanoparticles are usually engineered by chemical reactions in gas phase or solutions^2^,^3^, possible exposure routes appear to be foreseeable. Inhalation of airborne nanoparticles is a major exposure scenario^30^, and an interim report on risk assessment of C_60_ proposed the acceptable exposure concentration of 0.8 mg/m^3^ as respirable dust in working environments^31^.

The supposition is not always true, however. Glover et al. showed nanoparticles were spontaneously generated from macroscopic silver and copper objects when they were simply in contact with surfaces in a humid air^32^. Proposed chemical mechanism involves surface oxidation with ambient oxygen and adsorbed water, diffusion of metal ions from the parent particle in the adsorbed water layer, and nucleation via chemical and/or photochemical reduction of the ions. Their findings imply that macroscopic objects can be a potential source of incidental nanoparticles in the environment, and that humans have long been in direct contact with these nanomaterials without being noticed^32^.

Our results show there exists a mechanical pathway for similar incidental generation of nanoparticles from bulk C_60_ solids, and suggest highly unconventional exposure routes to nanoparticles of C_60_. For example, if one rubs a spillage of C_60_ solids on a lab bench with a bare fingertip, he/she may be exposed inadvertently to risk of dermal uptake and inhalation of C_60_ nanoparticles, even though he/she presumes handling bulk solids of C_60_ and does not foresee exposure to nanoparticles. Similar situations may be encountered commonly in research laboratories or manufacturing facilities that use C_60_, or during disposal of products that are deemed to contain solid C_60_ of macroscopic size. A possibility was also suggested that commercial solid C_60_ contains nanoparticles that are generated by friction of solid particles during production, storage, or transportation^9^.

Although formation of nanoparticles by simple pulverisation is not known for other materials to the best of our knowledge, it is worth mentioning that nanoparticles are produced by simple pulverisation when mechano-chemical reactions are involved. For example, Rao et al. demonstrated that nearly pure Ag_9_ quantum clusters were prepared by hand-grinding AgNO_3_ and mercaptosuccinic acid solids followed by reduction by NaBH_4_ using a mortar and pestle^33^. Thus, detailed understandings of underlying mechanisms behind the mechanical and/or mechano-chemical generation of nanoparticles form bulk solids, which still remain largely unclear, are crucial in characterizing environmental and health impacts of nanoparticles.

## Methods

### Materials

C_60_ (> 99.9% pure) was obtained from Tokyo Kasei, Co., Ltd. (Tokyo, Japan), and used as received. Sodium dodecyl sulphate (SDS) was purchased from Nacalai Tesque, Inc. (Kyoto, Japan). Millipore water was used throughout the work.

### Scanning electron microscopy

After rubbing C_60_ between glass microscope slides, one slide was held, with the side with C_60_ down, above a conductive double-sided tape that was mounted on a brass stub. The upper side of the slide was tapped with a lab spoon so that fine particles of rubbed C_60_ fell on the tape. Solid C_60_ at the mouth of a reagent bottle was collected in a same manner. The specimens were coated with osmium (estimated coating thickness, < 10 nm), and examined on a JSM-6700F (JEOL, Tokyo, Japan).

### High-resolution transmission electron microscopy

A drop of the dispersion of nanoparticles of C_60_ in water containing 1 wt% SDS was deposited on a carbon TEM grid. The surface of the carbon supporting film was subjected to hydrophilic treatment before the sample deposition. Crystals of SDS that precipitated after drying the specimen was removed with methanol. The specimen was air-dried again, and was examined on a Hitachi HF-2000 operating at 200 kV incident beam energy. The observations were performed at Nissan-Arc, Ltd. (Yokosuka, Japan).

### Dynamic light scattering

Average size of the C_60_ nanoparticle in the aqueous dispersion containing 1 wt% SDS was measured by dynamic light scattering on an FDLS-1200 (Otsuka Electronics Co., Ltd., Osaka, Japan) equipped with a solid-state laser (λ = 532 nm, 100 mW). The measurements were done at 25.0 ± 0.1°C and at a fixed scattering angle of 90°. Average hydrodynamic diameter was calculated by using a cumulant method^34^, while CONTIN^35^ was employed to obtain a size distribution. The dispersion was 100-fold diluted with water before the measurements.

## Author Contributions

S.D. and S.M. conceived and designed the research. S.D., S.M., H.S. and Y.N. performed the experiments and analysed the data. S.D. wrote the paper, and all authors reviewed the manuscript.

## References

[b1] ASTM Standard E2456, 2006 (2012), "*Standard terminology relating to nanotechnology*," ASTM International, West Conshohocken, PA, 2012, DOI: 10.1520/E2456-06R12.

[b2] Schmid G. *Nanoparticles: From theory to application* (Wiley-VCH, Weinheim, 2004).

[b3] Trindade T., O'Brien P. & Pickett N. L. Nanocrystalline semiconductors: Synthesis, properties, and perspectives. Chem. Mater. 13, 3843–3858 (2001).

[b4] Clement S. & Purutyan H. Narrowing down equipment choices for particle-size reduction. Chem. Eng. Prog. 98, 50–54 (2002).

[b5] Maa Y.-F. & Prestrelski S. J. Biopharmaceutical powders: Particle formation and formulation considerations. Curr. Pharm. Biotechnol. 1, 283–302 (2000). 11469385 10.2174/1389201003378898

[b6] Koch C. C. Materials synthesis by mechanical alloying. Annu. Rev. Mater. Sci. 19, 121–143 (1989).

[b7] Basset D., Matteazzi P. & Miani F. Designing a high energy ball-mill for synthesis of nanophase materials in large quantities. Mater. Sci. Eng. A. 168, 149–152 (1993).

[b8] Deguchi S., Mukai S., Yamazaki T., Tsudome M. & Horikoshi K. Nanoparticles of fullerene C_60_ from engineering of antiquity. J. Phys. Chem. C 114, 849–856 (2010).

[b9] Deguchi S., Mukai S., Tsudome M. & Horikoshi K. Facile generation of fullerene nanoparticles by hand grinding. Adv. Mater. 18, 729–732 (2006).

[b10] Deguchi S., Alargova R. G. & Tsujii K. Stable dispersions of fullerenes, C_60_ and C_70_, in water. Preparation and characterization. Langmuir 17, 6013–6017 (2001).

[b11] McCready D. E. & Alnajjar M. S. Powder data for buckminsterfullerene, C_60_. Powder Diffr. 9, 93–95 (1994).

[b12] Jakubczyk D. *et al.* Study of microscopic properties of water fullerene suspensions by means of resonant light scattering analysis. J. Phys. D: Appl. Phys. 37, 2918–2924 (2004).

[b13] Brant J., Lecoanet M., Hotze M. & Wiesner M. Comparison of electrokinetic properties of colloidal fullerenes (n-C_60_) formed using two procedures. Environ. Sci. Technol. 39, 6343–6351 (2005). 16190186 10.1021/es050090d

[b14] Deguchi S. & Mukai S. Top-down preparation of dispersions of C_60_ nanoparticles in organic solvents. Chem. Lett. 35, 396–397 (2006).

[b15] Deguchi S., Yamazaki T., Mukai S., Usami R. & Horikoshi K. Stabilization of C_60_ nanoparticles by protein adsorption and its implications for toxicity studies. Chem. Res. Toxicol. 20, 854–858 (2007). 17503852 10.1021/tx6003198

[b16] Kato H. *et al.* Characterization of fullerene colloidal suspension in a cell culture medium for in vitro toxicity assessment. Mol. BioSyst. 6, 1238–1246 (2010). 20414485 10.1039/c002364g

[b17] Yannoni C. S., Johnson R. D., Meijer G., Bethune D. S. & Salem J. R. ^13^C nmr study of the C_60_ cluster in the solid state: Molecular motion and carbon chemical shift anisotropy. J. Phys. Chem. 95, 9–10 (1995).

[b18] Dresselhaus M. S., Dresselhaus G. & Eklund P. C. Science of fullerenes and carbon nanotubes. (Academic Press, 1996).

[b19] Labille J. *et al.* Hydration and dispersion of C_60_ in aqueous systems: The nature of water−fullerene interactions. Langmuir 25, 11232–11235 (2009). 19725560 10.1021/la9022807

[b20] Luengo G., Campbell S. E., Srdanov V. I., Wudl F. & Israelachvili J. N. Direct measurement of the adhesion and friction of smooth C_60_ surfaces. Chem. Mater. 9, 1166–1171 (1997).

[b21] Colvin V. L. The potential environmental impact of engineered nanomaterials. Nat. Biotechnol. 21, 1166–1170 (2003). 14520401 10.1038/nbt875

[b22] Oberdörster E. Manufactured nanomaterials (fullerenes, C_60_) induce oxidative stress in the brain of juvenile largemouth bass. Environ. Health Perspect. 112, 1058–1062 (2004). 15238277 10.1289/ehp.7021 PMC1247377

[b23] Lewinski N., Colvin V. & Drezek R. Cytotoxicity of nanoparticles. Small 4, 26–49 (2008). 18165959 10.1002/smll.200700595

[b24] Klaine S. J. *et al.* Nanomaterials in the environment: Behavior, fate, bioavailability, and effects. Environ. Toxicol. Chem. 27, 1825–1851 (2008). 19086204 10.1897/08-090.1

[b25] Johnston H. J., Hutchison G. R., Christensen F. M., Aschberger K. & Stone V. The biological mechanisms and physicochemical characteristics responsible for driving fullerene toxicity. Toxicol. Sci. 114, 162–182 (2010). 19901017 10.1093/toxsci/kfp265

[b26] Andrievsky G. V., Kosevich M. V., Vovk O. M., Shelkovsky V. S. & Vashchenko L. A. On the production of an aqueous colloidal solution of fullerenes. J. Chem. Soc., Chem. Commun. 1281–1282 (1995).

[b27] Endoh S., Maru J., Uchida K., Yamamoto K. & Nakanishi J. Preparing samples for fullerene C_60_ hazard tests: Stable dispersion of fullerene crystals in water using a bead mill. Adv. Powder Technol. 20, 567–575 (2009).

[b28] Alvarez P. J. J., Colvin V., Lead J. & Stone V. Research priorities to advance eco-responsible nanotechnology. ACS Nano 3, 1616–1619 (2009). 21452862 10.1021/nn9006835

[b29] Maynard A. D. *et al.* Safe handling of nanotechnology. Nature 444, 267–269 (2006). 17108940 10.1038/444267a

[b30] Oberdörster G., Oberdörster E. & Oberdörster J. Nanotoxicology: An emerging discipline evolving from studies of ultrafine particles. Environ. Health Perspect. 113, 823–839 (2005). 16002369 10.1289/ehp.7339 PMC1257642

[b31] Morimoto Y. *et al.* Hazard assessments of manufactured nanomaterials. J. Occup. Health. 52, 325–334 (2010). 20972355 10.1539/joh.r10003

[b32] Glover R. D., Miller J. M. & Hutchison J. E. Generation of metal nanoparticles from silver and copper objects: Nanoparticle dynamics on surfaces and potential sources of nanoparticles in the environment. ACS Nano 5, 8950–8957 (2011). 21985489 10.1021/nn2031319

[b33] Rao T. U. B., Nataraju B. & Pradeep T. Ag_9_ quantum cluster through a solid-state route. J. Am. Chem. Soc. 132, 16304–16307 (2010). 21033703 10.1021/ja105495n

[b34] Koppel D. E. Analysis of macromolecular polydispersity in intensity correlation spectroscopy: The method of cumulants. J. Chem. Phys. 57, 4814–4820 (1972).

[b35] Provencher S. W. Inverse problems in polymer characterization: Direct analysis of polydispersity with photon correlation spectroscopy. Makromol. Chem. 180, 201–209 (1979).

